# ECG Signal Analysis for Detection and Diagnosis of Post-Traumatic Stress Disorder: Leveraging Deep Learning and Machine Learning Techniques

**DOI:** 10.3390/diagnostics15111414

**Published:** 2025-06-02

**Authors:** Parisa Ebrahimpour Moghaddam Tasouj, Gökhan Soysal, Osman Eroğul, Sinan Yetkin

**Affiliations:** 1 Biomedical Device Technology, Vocational School of Health Services, Ankara Medipol University, Ankara 06050, Turkey; parisa.ebrahimpour@ankaramedipol.edu.tr; 2 Department of Electrical and Electronics Engineering, Ankara University, Ankara 06830, Turkey; 3 Department of Biomedical Engineering, TOBB University of Economics and Technology, Ankara 06510, Turkey; erogul@etu.edu.tr; 4 Department of Psychiatry, Ankara Gülhane Health Application and Research Hospital, University of Health Sciences, Ankara 34668, Turkey; sinan.yetkin@sbu.edu.tr

**Keywords:** continuous wavelet transform, deep learning, electrocardiogram, machine learning, PTSD

## Abstract

**Background:** Post-traumatic stress disorder (PTSD) is a serious psychiatric condition that can lead to severe anxiety, depression, and cardiovascular complications if left untreated. Early and accurate diagnosis is critical. This study aims to develop and evaluate an artificial intelligence-based classification system using electrocardiogram (ECG) signals for the detection of PTSD. **Methods:** Raw ECG signals were transformed into time–frequency images using Continuous Wavelet Transform (CWT) to generate 2D scalogram representations. These images were classified using deep learning-based convolutional neural networks (CNNs), including AlexNet, GoogLeNet, and ResNet50. In parallel, statistical features were extracted directly from the ECG signals and used in traditional machine learning (ML) classifiers for performance comparison. Four different segment lengths (5 s, 10 s, 15 s, and 20 s) were tested to assess their effect on classification accuracy. **Results:** Among the tested models, ResNet50 achieved the highest classification accuracy of 94.92%, along with strong MCC, sensitivity, specificity, and precision metrics. The best performance was observed with 5-s signal segments. Deep learning (DL) models consistently outperformed traditional ML approaches. The area under the curve (AUC) for ResNet50 reached 0.99, indicating excellent classification capability. **Conclusions:** This study demonstrates that CNN-based models utilizing time–frequency representations of ECG signals can effectively classify PTSD with high accuracy. Segment length significantly influences model performance, with shorter segments providing more reliable results. The proposed method shows promise for non-invasive, ECG-based diagnostic support in PTSD detection.

## 1. Introduction

PTSD is a complex and multidimensional psychiatric disorder that develops following a traumatic event that threatens the individual’s life or causes a deep emotional shock. This condition manifests itself with symptoms such as flashbacks, nightmares, intense anxiety, and uncontrollable thoughts that arise in connection with the traumatic event. PTSD not only affects individual psychological health but also has serious adverse effects on social relationships, work life, and general functioning [1]. According to the World Health Organization’s World Mental Health Surveys, 70.4% of people worldwide have been through at least one traumatic event, with an average of 3.2 traumas per person [2]. Traumas involving interpersonal violence are the factors that increase the risk of PTSD the most. The highest burden of PTSD is caused by traumas such as war-related trauma [3], rape, sexual assault, stalking, and the sudden death of a loved one [4,5].

PTSD shows up in more than just psychological issues. However, it is also considered a significant risk factor for the development of serious physical health problems, such as cardiovascular disease (CVD) [6]. In a study [7], the relationship between PTSD and CVD was examined, and it was determined that the co-occurrence of these two conditions exhibited a supra-additive interaction. PTSD is associated with cardiometabolic diseases, anxiety, alcohol use disorder, and depression. However, the link between PTSD and cardiometabolic diseases and the effects of socioeconomic status and other comorbid conditions on this relationship is unclear [8]. In a study conducted on epidemics, PTSD and psychological distress were examined in patients who had COVID-19. The decrease in mortality rates and the effect of vaccines reduced psychological distress. However, there was no significant decrease in PTSD rates between waves. Comorbidities of patients were considered a significant risk factor for the development of PTSD [9]. A study was conducted in the United States that examined the relationship between PTSD and coronary heart disease (CHD) in young soldiers who had war experiences. The intense stress experienced due to war increased the risk of CHD. PTSD has not been directly linked to CHD, considering the effects of depression and anxiety. However, it has been stated that stress can be effective in the development of CHD in a short period. These studies and many more in the literature show that PTSD and cardiac functioning are related [10].

An ECG is a method that measures the electrical activity of the heart and is used in its diagnosis and screening. This non-invasive technique is a reliable and effective tool for assessing heart health [11]. ECG signals are used to monitor and diagnose abnormalities in the heart. These signals are defined by parameters such as PR, QRS, QT intervals, ST segment [12], heart rate (HR) [13], and heart rate variability (HRV) [14]. Some artifacts may cause difficulties when recording ECG signals, especially in determining ST segments and QRS waveforms; noise interferences may cause significant problems. One of these interferences, motion artifact, is caused by the movement and friction between the body and the electrode surface. The spectral range of motion artifact is very close to ECG signals, which makes it difficult to suppress such noise [15], power line interference (PLI), and baseline wander (BW) [16]. Different methods to eliminate these artifacts can be divided into two main classes: adaptive and non-adaptive approaches [17]. Although obtaining information via ECG is economical and accessible, collecting and processing these signals correctly is equally important.

Artificial intelligence approaches such as ML and DL have made enormous strides in healthcare technologies. These methods have achieved high feature extraction and classification accuracy from extensive, complex data. In this context, researchers have used ML and DL methods in the literature to detect PTSD through physiological signals. Since CNN, one of the DL methods, can automatically extract features from data, there is no need for manual feature extraction or selection processes, as in traditional ML methods. This is an essential advantage of CNN [18]. Recent studies have shown that 1D CNNs are quite effective in real-time ECG monitoring and anomaly detection [19]. Unlike traditional methods, 2D CNNs integrated with time–frequency analysis techniques such as CWT can extract both temporal and spectral features simultaneously. This approach increases the model’s performance by effectively representing the complex structure of ECG signals in different dimensions. Considering the success of CNNs in various biomedical applications, studies have shown that these models exhibit strong performance in detecting different cardiovascular diseases from ECG signals [20].

Literature studies show that models including CNNs, random forests, and SVMs recognize PTSD symptoms effectively [21]. Although conventional ML approaches such as SVMs are still extensively applied, DL approaches are becoming increasingly popular since they can learn complicated patterns and demand less feature engineering [22]. Models like random forest and XGB are reportedly more explainable than DL techniques [23]. Other research looks at several methods to identify PTSD, including the analysis of circadian rhythm alterations using wearable data [24], analysis of neuroimaging data, including fMRI, MEG, and EEG [25,26], and analysis of voice and text data from clinical interviews [27]. These few strategies point to a spectrum of possible data sources and machine learning techniques that fit PTSD.

ECG signals have a time-varying, non-stationary structure and can show sudden changes. Time–frequency methods are quite effective for the analysis of such signals. In particular, the wavelet transform can capture sudden changes in the signal by presenting both time and frequency information at different scales. In this way, slow and fast changes can be better distinguished. In our study, we evaluated the performance of these methods on ECG signals by evaluating the most up-to-date transfer learning models for PTSD detection and classification. Consistent with these objectives, the study encompasses the following aims:

- This study introduces a novel, state-of-the-art approach for the classification of PTSD from ECG signals, which has not been addressed in previous research using either DL or ML methods.
- To improve feature representation, we optimized the performance of CNN architectures using scalogram-based time–frequency images. This method increased the ability to capture complex patterns associated with PTSD more effectively.
- 5-fold cross-validation (K-fold cross-validation) was used to address the difficulties arising from the datasets. This method increased the model’s generalization ability and provided a more balanced and robust performance.
- Compared with traditional ML methods, a clear superiority of DL models was observed.
- The method in this study achieved higher accuracy rates than other DL models and traditional ML methods. These results provide information that can contribute to the development of more effective diagnostic tools for detecting psychological disorders.

## 2. Materials and Methods

**Figure 1:**
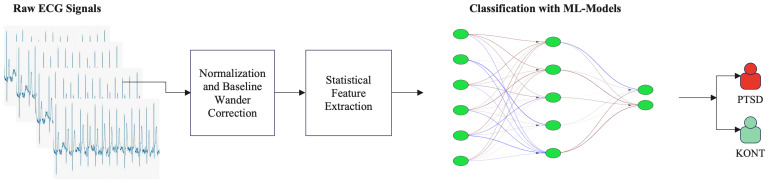
ML classification process.

In the first part of the study, ML was used to classify the dataset. Figure 1 shows the classification process using ML to discriminate between the PTSD and control groups. Initially, raw ECG signals from 20 individuals with PTSD and 20 individuals in the control group were processed in a time series fashion. The signals went through preprocessing, which included normalization and baseline drift correction. Then, a statistical feature extraction step was applied to obtain meaningful features (e.g., mean, standard deviation, variance) from ECG signals. These features were separated into training and test sets according to ML models with the 5-fold cross-validation method. Finally, different ML models were trained to classify individuals as belonging to the PTSD or control group.

**Figure 2:**
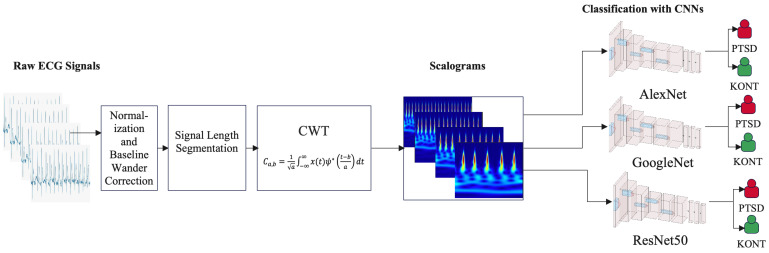
DL classification process.

DL was utilized in the second part of this study, and a mechanism for classifying ECG data using the transfer learning method was developed. Transfer learning decreases training time while increasing performance by allowing previously trained models to adapt to new datasets. Powerful CNN models such as AlexNet, GoogLeNet, and ResNet50 were utilized in this study. The block diagram in Figure 2 depicts how the operation begins with processing raw ECG signals. First, the signals are normalized, and baseline drift is corrected. The signals are then divided into 5, 10, 15, and 20-s segments to enable a more thorough analysis of the time-dependent effects. The segments are subjected to time–frequency analysis with the CWT method, which produces scalograms that reflect both the time and frequency components of the signal. These generated scalograms serve as input for CNN models. Model training and testing are carried out using the 5-fold cross-validation approach, ensuring the dataset is evaluated relatively. In the last stage, pre-trained models like AlexNet, GoogLeNet, and ResNet50 classify ECG signals using scalograms. This method aims to improve the classification performance by simultaneously extracting time–frequency information for PTSD and healthy control (KONT) groups.

### 2.1. Data Acquisition

The ECG signals used in this study were recorded at the Sleep Research Centre of Gülhane Education and Research Hospital between 2017 and 2022. The necessary ethical approval for the study was obtained from the Non-Interventional Scientific Research Ethics Committee (2024/25). The ECG recordings used in this study were collected from a collaborating hospital under the supervision of medical professionals. Each subject was evaluated by a licensed psychiatrist who confirmed the PTSD or healthy control status in accordance with DSM-5 criteria. Additionally, a certified cardiologist reviewed and confirmed the quality and validity of the ECG signals. The dataset consists of the records of 20 individuals diagnosed with PTSD by expert psychiatrists and a control group of 20 individuals who were determined to be healthy and had no history of psychiatric or cardiologic diagnosis based on physician evaluations and expert opinions. The impedance of all electrodes was kept below 10 kΩ, and the signals were digitized with a sampling frequency of 200 Hz. In addition, the recorded signals were filtered with a band-pass filter in the 0.1–70 Hz range to eliminate unwanted noise and artifacts. The dataset is based on 5 min ECG recordings. Each recording contains 60,000 samples. The segmentation process divided these signals into 5, 10, 15, and 20 s segments to create scalogram images. Thanks to this process, the total number of images increased significantly. The number of samples and scalograms for each segment length are given in Table 1.

This table shows that our dataset offers sufficient diversity and size to train DL models. The difference in segment lengths provided an advantage in comparing the overall performance of the models.

### 2.2. Signal Preprocessing

#### 2.2.1. Normalization and Baseline Wander Correction

BW is a low-frequency artifact in the ECG signal resulting from respiration, variations in electrode impedance, or motion. A high-pass zero-phase filter has been constructed utilizing a finite impulse response (FIR) methodology, defined by a cut-off frequency of 0.5 Hz. Consequently, low-frequency noise in the data has been successfully eradicated. Following error correction, Z-score normalization was applied to the data. Z-score normalization is a fundamental preprocessing method employed to standardize signal amplitudes. This approach enables the study of diverse signals within a uniform framework by removing amplitude variations among them. This renders the varied signal data similar and produces more uniform results in analysis procedures. In Equation (1), *x* denotes the input signal, μ signifies the estimated mean value of the signal, and σ indicates the estimated standard deviation of the signal.

(1) $$z=\frac{x-\mu }{\sigma }$$

#### 2.2.2. One-Dimensional ECG Signal into a Two-Dimensional Image

The Continuous Wavelet Transform converts a one-dimensional ECG signal into a two-dimensional image, facilitating time–frequency analysis. CWT analyzes several frequency components within the signal at predetermined time intervals, elucidating the attributes of non-stationary transmissions. The resultant “scalogram” depicts the temporal variations in the signal’s frequency content. CWT facilitates the analysis of low-frequency components, including P and T waves, and temporal aspects like QRS complexes. A wavelet is a localized wave that concentrates its energy over time to analyze transient and non-stationary phenomena. Although it displays wave-like properties, it can perform simultaneous time and frequency analysis. This emphasizes the difference between a sinusoidal wave with boundless energy and a wavelet with infinite energy concentrated at a particular location [28]. A wavelet is a finite-duration waveform having a mean value of zero. It may be characterized as follows:

(2) $$\psi_{a,b}=\frac{1}{\sqrt{a}}\psi \left(\frac{t-b}{a}\right),\;a,b\in R$$

Here, *a* is called the dilation (scale) parameter stretch and compresses the wavelet size, and *b* is the translation (position) parameter that shifts the wavelet. The equation then gives the CWTs of signal *x(t)*:

(3) $$C_{a,b}=\frac{1}{\sqrt{a}}\int_{-\infty }^{\infty }x\left(t\right)\psi^{*}\left(\frac{t-b}{a}\right)dt$$

**Figure 3:**
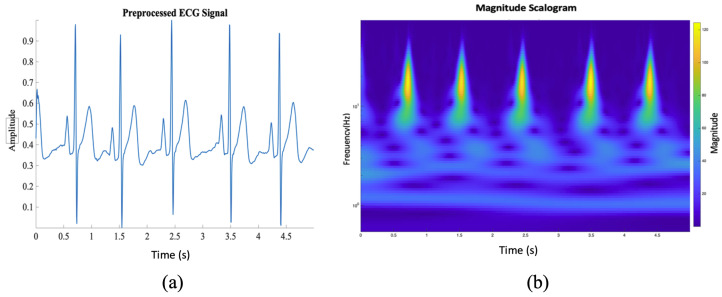
Conversion of a 5-s ECG segment into a scalogram using Continuous Wavelet Transform (CWT). (**a**) A sample 1D ECG signal extracted from a 5 s segment. (**b**) The corresponding 2D time–frequency representation (scalogram) was generated using the Morlet wavelet.

In this equation, *x(t)* is the input signal and $\psi^{*}$ is the wavelet function [29]. CWT can reveal the dynamic frequency characteristics of the signal with a smooth wavelet. This study will use this feature to obtain information from ECG signals. We preferred the Morlet wavelet by applying various scales and shifts represented by Equation (4). The Morlet wavelet derived from the Gaussian function has more advantages than other wavelets, such as Morse and Bump, because it provides high resolution. The parameter σ strongly influences the mother wavelet’s shape. Figure 3 shows the time–frequency analysis of the 5-s ECG signal analyzed with the Morlet wavelet.

(4) $$\psi_{\mathrm{Morlet}}\left(t\right)=\left(\cos(2\pi t)+i\sin(2\pi t)\right)e^{-\frac{t^{2}}{2\sigma^{2}}}$$

### 2.3. Performance Evaluation

Evaluating the performance of a model in binary classification tasks requires more than just knowing how many predictions are correct. Therefore, this study uses some comprehensive evaluation metrics derived from the confusion matrix to evaluate the adequacy of the proposed method. These metrics include accuracy, precision, recall (sensitivity), specificity, F1 score, Matthews correlation coefficient (MCC), and prevalence. Accuracy indicates the overall correct predictions. Precision indicates correctly predicted positive cases among all recorded positives, while recall measures the proportion of correctly identified true positives. Specificity can evaluate the capacity of the model to identify negative cases correctly. F1 reward combines precision and recall for false positives and false negatives into a single harmonic mean. MCC is a mixed metric that adds all the uncertainty of the confusion matrix and is especially useful when the class distributions are unbalanced. Finally, prevalence indicates the occurrence of true positive cases in the data. These criteria, defined in Equations (5)–(11), provide an interpretable assessment of the classification model in medical diagnoses where all predictions carry significant consequences.

(5) $$\mathrm{Accuracy}=\frac{TP+TN}{TP+TN+FP+FN}$$

(6) $$\mathrm{Precision}=\frac{TP}{TP+FP}$$

(7) $$\mathrm{Sensitivity}\;\left(\mathrm{Recall}\right)=\frac{TP}{TP+FN}$$

(8) $$\mathrm{Specificity}=\frac{TN}{TN+FP}$$

(9) $$F1\text{-}\mathrm{Score}=2\times \frac{\mathrm{Precision}\times \mathrm{Recall}}{\mathrm{Precision}+\mathrm{Recall}}$$

(10) $$M\;C\;C=\frac{\left(TP\times TN-FP\times FN\right)}{\sqrt{\left(TP+FP)(TP+FN)(TN+FP)(TN+FN\right)}}$$

(11) $$\mathrm{Prevalence}=\frac{TP+FN}{TP+TN+FP+FN}$$

## 3. Results

### 3.1. Machine Learning Classification

The selected statistical features represent a wide range of signal characteristics, including amplitude-based parameters such as peak value, root mean square (RMS), and mean; variation-based metrics like standard deviation (STD), skewness, and kurtosis; shape-related descriptors including shape factor, crest factor, clearance factor, and impulse factor; and signal quality-related indicators such as signal-to-noise ratio (SNR), signal-to-noise and distortion ratio (SINAD), and total harmonic distortion (THD). These features were implemented and computed in the MATLAB environment. These features were selected based on their established relevance in the biomedical signal processing literature, as they effectively capture both morphological and statistical characteristics of ECG signals in pathological versus control populations. Incorporating this comprehensive set ensures a robust representation of the ECG’s time-domain dynamics and statistical properties, significant in psychiatric disorder detection, where subtle waveform deviations may carry critical diagnostic value.

These features were selected based on their widespread use in biomedical signal analysis and their capacity to describe the ECG waveform’s statistical structure and dynamic behavior. Feature importance was further assessed using one-way ANOVA, revealing that although some features showed stronger discriminative power, excluding any subset led to a decline in classification accuracy. Therefore, all features were retained in the final model to ensure maximum diagnostic performance. A total of 13 time-domain statistical features were extracted from the ECG signals to characterize their morphological and amplitude-based dynamics. Table 2 summarizes the extracted features, their technical definitions, and their specific relevance in the context of ECG signal analysis for PTSD detection.

**Figure 4:**
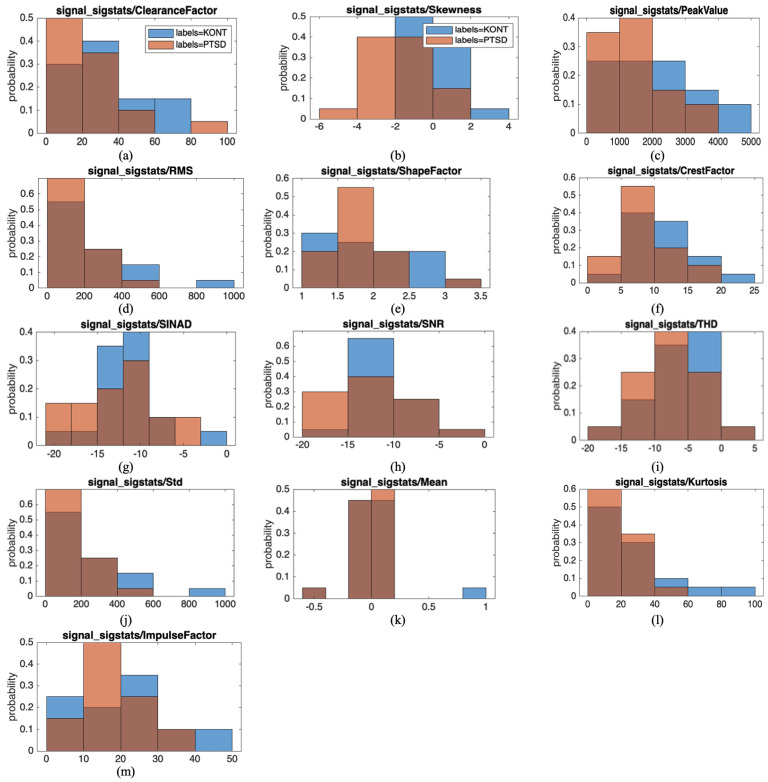
Statistical feature distributions of ECG signals for KONT (control) and PTSD groups: (**a**) clearance factor, (**b**) skewness, (**c**) peak value, (**d**) RMS, (**e**) shape factor, (**f**) crest factor, (**g**) SINAD, (**h**) SNR, (**i**) THD, (**j**) standard deviation, (**k**) mean, (**l**) kurtosis, and (**m**) impulse factor. Each subplot shows the probability distribution of the respective feature values, with blue bars representing the control group (KONT) and orange bars representing the PTSD group. The x-axis represents the feature value ranges, and the y-axis represents the probability density.

The distribution of 13 statistical features extracted from ECG signals is presented in Figure 4. The probability distributions demonstrate distinct morphological and signal quality differences between groups. Clearance factor shows a broader distribution in PTSD subjects, indicating increased detection of impulsive ECG morphology abnormalities. Skewness reveals asymmetric waveform patterns with positive values predominating in PTSD and negative values in controls, suggesting altered cardiac electrical conduction patterns. Peak value captures more extreme R-peak events in the PTSD group, reflecting potential changes in ventricular depolarization amplitude. Signal quality metrics (SINAD, SNR, and THD) exhibit group-specific patterns, with PTSD subjects showing different noise artifact characteristics and signal distortion profiles. Shape-related features (shape factor and crest factor) indicate altered ECG waveform morphology and QRS complex transitions in PTSD. Statistical measures (RMS, standard deviation, kurtosis, and impulse factor) reveal differences in overall signal energy, variability, arrhythmic spike detection, and sensitivity to sudden cardiac events such as premature beats, collectively providing quantitative evidence of ECG alterations associated with PTSD pathophysiology.

RMS shows the average power level of the waveforms of the signal, whereas the RMS shows a wider distribution in the PTSD group, indicating that the amplitude variability of the signals in this group is high, and their energy levels differ. STD shows a broader distribution in the PTSD group, reflecting the amplitude changes of the signals. The fact that the PTSD group has higher values in shape features such as the shape factor and crest factor indicates differences in the geometric structure of the signals. Regarding SINAD and SNR, the control group has a more significant dispersion that approaches negative values, showing that the signals are noise-sensitive. In the PTSD group, the mean values shift towards positive, while in the control group, the distribution is more balanced. Kurtosis had higher values in the PTSD group, reflecting differences in the extreme values of the signals. Finally, the impulse factor showed a higher dispersion in the PTSD group, revealing differences in the prominence of the signal peaks. All these statistical features are distinguishing features that may play a critical role in the classification of the PTSD and control groups. Table 3 examines the performance of various ML models for categorizing ECG signals. Linear discriminant analysis (LDA) performed best, with a 72.50% accuracy rate and balanced metrics (precision, recall, specificity, and F1 score), making it the most acceptable method for classification. The ensemble model correctly identified negative classes with 80.00% specificity. However, its low recall made it ineffective in detecting positive classes.

The SVM and KNN (cubic) models both achieved 65% accuracy; however, the SVM model had a low recall, indicating its inability to effectively detect positive cases. The trilayered neural network struggled with classifying negative samples due to its low specificity, despite achieving a high recall rate, and, thus, requires further optimization. Although naïve Bayes showed the lowest performance with 57.50% accuracy, it may still be applicable in specific scenarios where moderate metric scores are acceptable. LDA performed better than the other traditional methods in terms of overall metric consistency. When examining the MCC, which ranges from [−1 to +1], all models, including LDA, produced MCC values below 0.5, indicating a limited agreement between predicted and true class labels. These results suggest that while LDA shows relatively better performance, traditional ML methods, overall, are not sufficiently effective for this classification task.

### 3.2. Deep Learning Classification

In the preprocessing phase, ECG signals were divided into four different segment lengths (5 s, 10 s, 15 s, and 20 s). These segments were converted into two-dimensional time–frequency scalogram images. As a result of this process, respectively, 2400, 1200, 800, and 600 time–frequency images were obtained for each segment.

The input dimensions of each of the pre-trained deep networks used in the MATLAB environment are defined differently: 227 × 227 × 3 for AlexNet, and 224 × 224 × 3 for GoogLeNet and and ResNet50. Therefore, the data were processed separately to be suitable for the input dimensions required by each model and obtained directly from these dimensions. This difference is due to the input format that each architecture expects during training. To prevent the loss of critical information, images for each network are generated separately from the original data. Thus, essential features are not distorted during resolution change.

Cross-validation is a method used to evaluate the performance of a model more reliably and generally. Its primary purpose is to prevent overfitting and increase generalization ability by testing the model’s success on different examples in the dataset. This approach partitioned the dataset into five equal-sized subgroups. In each iteration, one group was utilized as test data, while the other four were used for training, and the procedure was repeated five times. Model accuracy, precision, and sensitivity were calculated for each fold, and the average of these values was used to determine the model’s overall performance.

Table 4 shows the training parameters. A binary classification structure was preferred in the model. The mini-batch size was determined as 20, and the maximum epoch number was defined as 8. “Stochastic Gradient Descent with Momentum” (SGDM) was used as the optimization algorithm, which allows the model to learn faster and more stably by reducing oscillations while optimizing its parameters [43]. The learning rate is set to 0.0001, ensuring the model’s weight updates occur in small increments. The validation frequency is set to 10, meaning the model is evaluated on the validation dataset every ten steps.

This study used DL models (AlexNet, GoogLeNet, and ResNet50) pre-trained with the ImageNet dataset with transfer learning and fine-tuning methods. In all three models, only the last three layers were changed and reconstructed as two classes (PTSD and control). This approach provided effective results without the need for a large amount of data and reduced the risk of overfitting.

Table 5 presents the architectural specifications and training characteristics of the three CNN models used in this study. AlexNet contains 60.9 million learnable parameters across 25 layers, while GoogLeNet is the most compact with 5.9 million parameters distributed over 144 layers. ResNet50 has 25.6 million parameters spanning 177 layers. All models underwent transfer learning with the final three layers replaced for binary PTSD classification. Training time varied significantly across models and segment lengths. ResNet50 required the longest training time, ranging from 130 ± 5 min for 5 s segments to 32 ± 5 min for 20 s segments per fold. GoogLeNet showed moderate training times (55 ± 5 to 13 ± 5 min), while AlexNet was the most efficient (29 ± 2 to 7 ± 2 min). The inverse relationship between segment length and training time reflects the increased number of samples generated from shorter segments, requiring more computational resources for model optimization.

**Figure 5:**
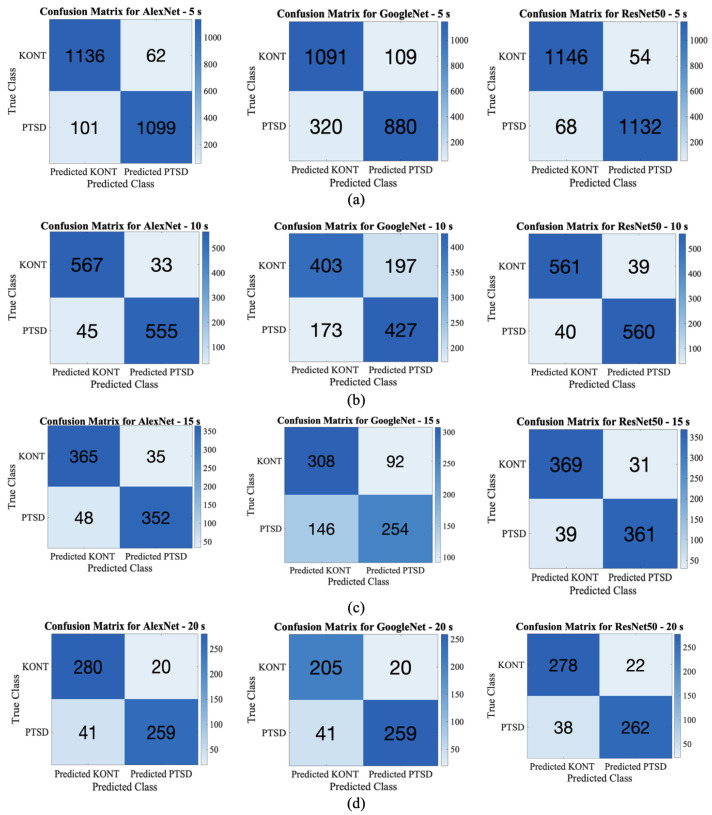
Confusion matrices from 5-fold cross-validation applied to DL models (AlexNet, GoogLeNet, and ResNet50) for PTSD vs. control classification. Results are shown for four ECG segment lengths: (**a**) 5 s, (**b**) 10 s, (**c**) 15 s, and (**d**) 20 s. Each matrix reports true positives (TP), false negatives (FN), true negatives (TN), and false positives (FP), enabling detailed evaluation of model performance.

Figure 5 illustrates the 5-fold cross-validation confusion matrices obtained from the classification of PTSD and control groups using AlexNet, GoogLeNet, and ResNet50 across four different signal segment lengths (5 s, 10 s, 15 s, and 20 s). Each matrix presents true negatives (TN), false positives (FP), false negatives (FN), and true positives (TP), allowing for a detailed interpretation of model performance. Figure 5a displays the results for the 5 s segments. ResNet50 achieved the highest classification accuracy, with fewer false negatives and false positives. AlexNet also performed well, while GoogLeNet exhibited a noticeably higher false negative rate, indicating difficulty in identifying PTSD samples correctly. Figure 5b represents the 10 s segments. As the segment length increased, GoogLeNet’s performance significantly declined, with more false negatives. ResNet50 continued to show robust performance with high true positive and true negative rates, indicating its resilience to changes in segment length. Figure 5c,d show the results for 15 and 20 s segments, respectively. All three models experienced a decline in accuracy compared to the 5 s segment condition. This performance drop can be attributed to reduced temporal resolution and increased complexity in longer segments, which may obscure critical time-localized features. ResNet50 remained the most consistent model despite this degradation.

The DL models employed in this study were evaluated using multiple measures for varying segment lengths. According to Table 6, the ResNet50 model performed the best across all segment lengths. In 5 s segments, it outperformed other models with 94.92% accuracy, 95.45% precision, 94.33% sensitivity, 95.50% specificity, and 94.89% F1 score. This demonstrates ResNet50’s ability to discriminate between positive and negative classifications accurately. The AlexNet model, on the other hand, produced successful results with high accuracy (93.21% and 93.50%) and an F1 score (93.10% and 93.43%), particularly in 5 and 10 s segments. Still, performance declined slightly as segment length rose. In contrast, GoogleNet showed lower accuracy (82.1–67.33%) and F1 scores in all segment lengths compared to other models. The low sensitivity and specificity values, especially in the 10, 15, and 20 s segments, indicate that GoogleNet is less reliable in classification.

When we examine the MCC, we observe that it provides a more balanced evaluation of the model’s performance. Unlike the accuracy metric, MCC takes into account all elements of the confusion matrix (TP, TN, FP, and FN) and is particularly useful in imbalanced datasets [44]. The MCC value ranges from −1 to +1, where +1 indicates perfect prediction, 0 means random prediction, and −1 shows total disagreement between predictions and actual values. In our study, the ResNet50 model with 5 s segments achieved the highest MCC score of 0.8983, followed by AlexNet-5s with 0.8645, while GoogLeNet-5s showed the lowest MCC performance with 0.6526. Although the highest accuracy (94.92%) was also achieved by ResNet50-5s, the slightly lower MCC value reflects that some misclassifications still occurred, which MCC captures more effectively than accuracy. Overall, both the high accuracy and MCC confirm that ResNet50-5s is the most successful DL architecture for distinguishing PTSD and control groups, especially in short-duration ECG segments.

### 3.3. ROC Performance Evaluation

The receiver operating characteristic (ROC) curve is a critical evaluation tool for determining model performance [45]. The true positive rate (TPR) and false positive rate (FPR) are computed, and TPR is graphed against FPR. Comparing classifiers using ROC curves can be difficult due to the lack of a single scalar value. Therefore, the area under the ROC curve (AUC) metric is used to quantify the area under the ROC curve. This provides a scalar value that represents the expected performance of the classifier and makes it easier to compare different models. The ROC curves of the DL models employed in our study were utilized to assess the classification performance between the PTSD and control groups. In Table 7, the ResNet50 model showed the best performance with a high AUC value of 0.991 in 5 s segments. This indicates that the model is an almost perfect classifier and maximizes the actual positive rate compared to the false positive rate. When compared to other models, ResNet50 is seen to exhibit superior performance in a wide threshold range. AlexNet, on the other hand, attracted attention with its high AUC values, especially in the 5 s and 10 s segments. AUC values were used as an effective metric to compare the overall success of the models, and it was concluded that ResNet50 was the most suitable model for this study.

**Figure 6:**
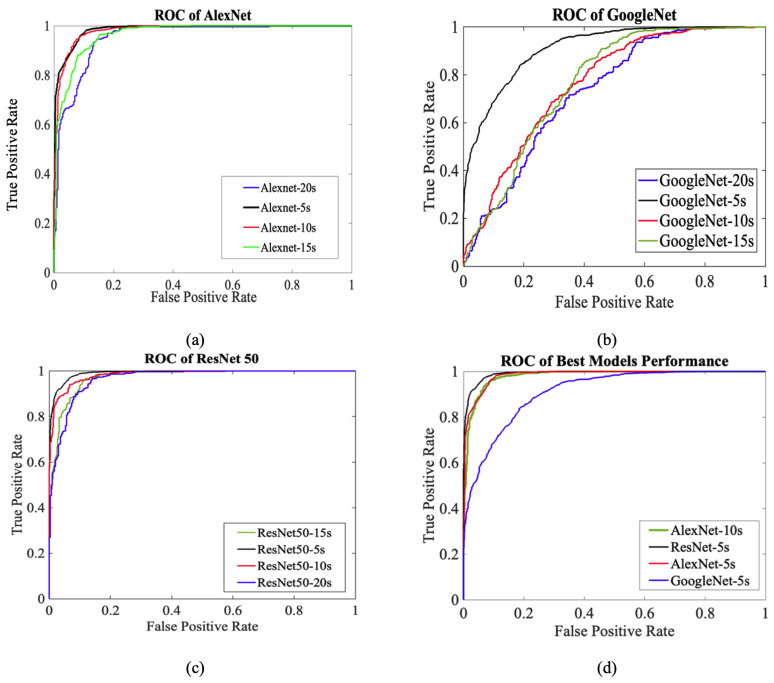
ROC curves of the CNN-based DL models evaluated across different ECG segment lengths. (**a**) ROC performance of the AlexNet model using 5 s, 10 s, 15 s, and 20 s segments. (**b**) ROC performance of the GoogLeNet model with varying segment lengths. (**c**) ROC curves for the ResNet50 model using the same segment durations. (**d**) Comparison of the best-performing models across all architectures based on their optimal segment lengths.

Figure 6 illustrates how the classification performance of the CNN-based models (AlexNet, GoogLeNet, and ResNet50) changes depending on the ECG segment length used for training. Each subfigure compares different segment durations (5 s, 10 s, 15 s, and 20 s) for a single model. Subfigure (d) highlights the best-performing configuration of each model to emphasize which combination of architecture and segment length yielded the highest classification capability. Among all, ResNet50 with 5 s segments achieved the highest AUC, demonstrating its superior ability in distinguishing between PTSD and control classes.

These ROC curves visualize the impact of segment length on model performance. The effect of segment length on model performance can be evaluated in terms of time–frequency representation, signal stationarity properties, and the model learning process. Since scalograms are based on time–frequency analysis, as segment length increases, time resolution decreases, and significant short-term changes may be lost. ECG signals are non-stationary; therefore, long segments may contain different rhythmic changes, making it difficult for the model to learn distinct patterns. In addition, shorter segments can increase the generalization ability by allowing the model to be trained on more data. For DL models, very long segments can cause unnecessary complexity. Segments of 5 s are suitable for time resolution and amount of information, and support learning the most distinct features. In addition to visual inspection of ROC curves, the AUC value provides a quantitative measure to compare the performance of models. A higher AUC indicates a better ability of the model to distinguish between classes. This value reduces the trade-off between sensitivity (TPR) and specificity (1-FPR) to a single number and summarizes the overall classification success, making it easier to compare models.

## 4. Discussion

To the best of our knowledge, this study is the first to systematically investigate the use of both DL and classical ML approaches for the classification of PTSD based on ECG signals. Unlike previous studies focusing primarily on EEG or imaging modalities, our work utilizes the time–frequency properties of ECG signals through scalogram representations derived from CWT. This approach enables the extraction of both short- and long-term temporal patterns that are highly relevant to the physiological manifestations of PTSD. We also explore the performance of multiple pre-trained CNN models with transfer learning, optimized via 5-fold cross-validation to ensure robustness. Furthermore, we provide a comprehensive comparative analysis between DL and ML classifiers, highlighting the superior generalization capacity of deep models in this context. The outcomes of this study offer a novel direction for ECG-based psychological disorder detection and present promising implications for future clinical decision support systems.

In addition to these findings, the classification performance was further evaluated using the MCC, which provides a more informative and reliable measure, especially for imbalanced datasets. Among all methods, the ResNet50 model trained on 5 s segments achieved the highest MCC value of 0.8983, along with the highest accuracy rate of 94.92%, indicating a strong correlation between predicted and true labels as well as overall classification success. In contrast, classical ML models, including LDA, yielded MCC values below 0.5, underscoring their limitations in this task. These results indicate the robustness and clinical potential of DL models, particularly ResNet50, in distinguishing PTSD from the control group using ECG signals.

Table 8 summarizes the selected studies that used various biological cues and methods to diagnose PTSD. Different data were studied, including functional MRI (fMRI), speech signals, video and audio markers, pupil size measurements, and EEG. These evaluations showed different accuracy and AUC results when using DL and traditional ML methods. Most studies hardly utilized time–frequency analysis enough, or did not take full advantage of DL methods. Our study used CNN models to classify PTSD by analyzing ECG data through time–frequency representations made with CWT. This method has an essential advantage over past studies since it uses both DL and time–frequency analysis together. To distinguish pediatric PTSD, ref. [25] developed a DL model using resting-state fMRI data and graphic topological measurements. However, since fMRI is an imaging method that is costly, time-consuming, and difficult to transport, its clinical applicability is limited. The fact that ECG is more cost-effective, accessible, and suitable for real-time analysis shows that our method provides advantages in terms of practical use. The study [26] used resting EEG data for PTSD diagnosis. SVM yielded 70.37% accuracy and 0.85 AUC. The study was based on phase-locking values and network measurements. However, time–frequency analysis and DL methods were not used. On the other hand, our research used scalogram-based time–frequency features to show more of the data and achieved better results with DL models like AlexNet and ResNet. In a study conducted for PTSD diagnosis [27], a deep belief network (DBN) model and a transfer learning strategy were used together. In the study, frequency features belonging to three different categories were extracted from speech signals, and these features were combined with the DBN model. The method was tested on two different PTSD speech databases were taken from 26 patients. Compared to traditional methods, support vector machines (SVM) achieved only 57.68% accuracy, while the transfer learning strategy increased the success of the DBN model from 61.53% to 74.99%. This study also showed that the transfer learning method is a correct approach. Another study [46] introduced a DL-based method that analyzes facial expressions, movement parameters, speech prosody, and natural language content for the diagnosis of PTSD and major depressive disorder (MDD).

The DL model was able to identify PTSD with 90% accuracy and sadness with 86% accuracy. However, although this study used digital biomarkers, it relied on indirect observations rather than a direct physiological signal, such as an ECG. Our study provides a more objective and biophysical analysis using ECG data that directly measure heart activity. In addition, ECG recordings are more reliable and accessible in remote assessment processes since they are low-cost, portable, and suitable for continuous monitoring. In another study supporting the results [47], a CNN-based model was developed using pupillometry data for PTSD diagnosis and achieved 81.09% accuracy. Pupillometry offers a fast and accessible method to measure autonomic nervous system changes. However, a spectrogram was used as a time–frequency representation in the study. Our study preferred a scalogram because it is more sensitive to temporal changes and provides better time–frequency localization thanks to the wavelet transform. In this way, higher accuracy was achieved in PTSD classification over ECG signals, which shows the superiority of our method.

Another study in the literature [48] used deep transfer learning for PTSD diagnosis by analyzing EEG signals with VGG16. This method, mainly because CWT was better at both time and frequency resolution, was significant in diagnosing PTSD because it gave more detailed information than other spectral analysis methods. However, only one CNN model was tested, and segmentation was not done. Our study provided a more comprehensive analysis by comparing different CNN architectures and offered optimal data representation by evaluating different segment lengths. In addition, we achieved higher accuracy by using ECG, a more accessible technology than EEG, which increases our methodology’s clinical applicability. Existing research on PTSD detection primarily relies on EEG, fMRI, speech, or pupillometry data, as summarized in Table 8. These studies employ various classifiers on non-ECG signals, such as DL and traditional ML models. Therefore, a direct comparison with similar methodologies is not feasible. In contrast, our study proposes an ECG-based framework, which is more cost-effective, portable, and easily applicable in clinical environments. This study addresses a critical gap in the field by proposing and evaluating novel ECG-based classification approaches, thereby setting a foundation for future comparative research in PTSD detection using ECG signals.

## 5. Conclusions

In conclusion, this study is the first to explore PTSD classification using ECG signals with DL and ML, making it a novel contribution to the field. Unlike ML algorithms, DL models could automatically extract distinctive features from raw data. Traditional ML algorithms are usually based on predefined statistical features and cannot exhibit similar performance. The difficulties and time constraints encountered in manual feature extraction, especially for determining features such as QRS-complex detection, are eliminated by DL methods; this provides higher performance and efficiency. This study introduces a scalogram-based approach for the detection of PTSD from the ECG signals and compares conventional ML methods with DL techniques. The scalogram can contribute to a more robust and accurate classification framework by representing the signal’s time–frequency characteristics. This method is used for PTSD classification by transforming 1D ECG signals into 2D scalogram images. Initially, the time series signals were segmented into four different lengths. Subsequently, the CWT was applied to generate scalograms, effectively representing the time–frequency characteristics of the ECG signals as images. These scalograms were then used as input for DL algorithms, specifically CNNs. CNNs, renowned for their proficiency in image analysis, offer various pre-trained models capable of automatically extracting relevant features, eliminating the need for manual feature extraction and potentially leading to more robust and accurate classification outcomes. This study demonstrates the remarkable efficacy of the proposed scalogram-based approach, utilizing segmented 5-s ECG images and leveraging the power of AlexNet and ResNet50 DL architectures. These two models managed to show remarkable performance at 5 and 10 s ECG lengths. In this case, when we look at the ROC curve, it is observed that it provides the best AUC, and the most reliable result is that of ResNet50. This model achieved exceptional MCC, accuracy, specificity, recall, and F1 score performance across all metrics evaluated.

## 6. Future Studies

The results of this study showed acceptable performance in binary PTSD classification. However, future research should use multi-class classification techniques to distinguish between various mental illnesses with similar symptoms. Moreover, DL methods can be used for feature extraction and classification, and ML techniques can be used to classify these features. In particular, this approach can provide an effective alternative for extracting complex features from signals and using these features in classification. The use of different methods to obtain time–frequency-based 2D images can be investigated. In this study, CWT-based scalograms were used; however, by trying methods such as Stockwell transform, poincare, chirplet, short time Fourier transform, Choi–Williams, and AOK, better time–frequency resolution can be achieved, and details such as P and T waves can be captured more clearly [58,59].

## Figures and Tables

**Table 1:** Scalogram counts by segment length.

Segment Length	Samples per Scalogram	Scalograms per Record	Total Records	Total Scalograms
5 s	1000	60	40	2400
10 s	2000	30	40	1200
15 s	3000	20	40	800
20 s	4000	15	40	600

**Table 2:** Statistical features extracted from ECG signals.

Feature (No)	Description	Mathematical Formula
Clearance Factor [30]	Detects impulsive abnormalities in ECG morphology	$CF=\frac{\max(|x|)}{{\left(\frac{1}{N}\sum_{i=1}^{N}\sqrt{|x_{i}|}\right)}^{2}}$
Skewness [31]	Identifies asymmetric waveform patterns	$\mathrm{Skewness}=\frac{\frac{1}{N}\sum_{i=1}^{N}{\left(x_{i}-\bar{x}\right)}^{3}}{\sigma^{3}}$
Peak Value [32]	Captures extreme events like R-peaks	PeakValue=max(|x|)
RMS [33]	Represents overall signal energy	$RMS=\sqrt{\frac{1}{N}\sum_{i=1}^{N}x_{i}^{2}}$
Shape Factor [34]	Describes overall ECG waveform shape	$\mathrm{Shape}\;\mathrm{Factor}=\frac{RMS}{\frac{1}{N}\sum_{i=1}^{N}\left|x_{i}\right|}$
Crest Factor [35]	Indicates sharp transitions like QRS complexes	$\mathrm{Crest}\;\mathrm{Factor}=\frac{\max(|x|)}{RMS}$
SINAD [36]	Evaluates ECG signal quality, including noise artifacts	$\mathrm{SINAD}=10\log_{10}\left(\frac{\sum x_{i}^{2}}{\sum {\left(x_{i}-{\bar{x}}_{i}\right)}^{2}}\right)$
SNR [37]	Assesses clarity of the ECG signal	$\mathrm{SNR}=10\log_{10}\left(\frac{\sum x_{i}^{2}}{\sum noise_{i}^{2}}\right)$
THD [38]	Identifies signal distortions or repetitive noise patterns	$\mathrm{THD}=\frac{\sqrt{\sum_{h=2}^{H}V_{h}^{2}}}{V_{1}}\times 100\%$
Standard Deviation [39]	Detects variability in ECG waveforms	$\sigma =\sqrt{\frac{1}{N}\sum_{i=1}^{N}{\left(x_{i}-\bar{x}\right)}^{2}}$
Mean [40]	Baseline reference of ECG amplitude	$\mathrm{Mean}=\frac{1}{N}\sum_{i=1}^{N}x_{i}$
Kurtosis [41]	Detects sharp features like arrhythmic spikes	$\mathrm{Kurtosis}=\frac{\frac{1}{N}\sum_{i=1}^{N}{\left(x_{i}-\bar{x}\right)}^{4}}{\sigma^{4}}$
Impulse Factor [42]	Sensitivity to sudden spikes such as premature beats	$\mathrm{Impulse}\;\mathrm{Factor}=\frac{\max(|x|)}{\frac{1}{N}\sum_{i=1}^{N}\left|x_{i}\right|}$

**Table 3:** Performance comparison among ML models.

ML-Model	Accuracy (%)	Precision (%)	Recall (%)	Specificity (%)	F1-Score (%)	MCC	Prevalence (%)
Linear Discriminant	72.50	73.68	70.00	75.00	71.79	0.4505	0.5
Ensemble	70.00	75.00	60.00	80.00	66.67	0.4082	0.5
SVM (Linear)	65.00	68.75	55.00	75.00	61.11	0.3061	0.5
KNN (Cubic)	65.00	66.67	60.00	70.00	63.16	0.3051	0.5
Trilayered Neural Network	62.50	60.87	70.00	0.55	65.12	0.2528	0.5
Naïve Bayes	57.50	67.00	70.00	75.00	0.7179	0.1549	0.5

**Table 4:** Training parameters and their importance.

Parameter	Value/Setting	Description and Importance
Class Structure	Two Classes	The model is trained to classify data into two categories, enabling binary classification and distinguishing between two distinct groups.
Mini-Batch Size	20	The model processes 20 samples at a time for each weight update, balancing memory usage and training stability. Smaller batches introduce noise, improving generalization.
Max Epochs	8	The model iterates over the entire training dataset a maximum of 8 times, preventing overfitting by limiting dataset exposure.
Optimization Algorithm	SGDM	SGDM with Momentum accelerates learning and reduces oscillations during parameter optimization.
Learning Rate	0.0001	Controls the step size for weight updates during training. A small learning rate ensures stable and precise updates, avoiding overshooting the optimal solution.
Validation Frequency	10	The model is evaluated on the validation dataset every 10 training steps to monitor performance, detect overfitting, and ensure generalization.

**Table 5:** Model specifications and training parameters.

Model	Learnable Parameters (M)	No. of Layers (MATLAB)	Fine-Tuning	Segment Length (s)	Training Time (per Fold, min)
AlexNet	60.9	25	Final 3 layers replaced	5	29 ± 2
				10	21 ± 2
				15	14 ± 2
				20	7 ± 2
GoogLeNet	6.9	144	Final 3 layers replaced	5	55 ± 5
				10	30 ± 5
				15	18 ± 5
				20	13 ± 5
ResNet50	25.5	177	Final 3 layers replaced	5	130 ± 5
				10	60 ± 5
				15	41 ± 5
				20	32 ± 5

**Table 6:** Deep learning models performance results.

DL-Model	Accuracy (%)	Precision (%)	Recall (%)	Specificity (%)	F1-Score (%)	MCC	Prevalence (%)
AlexNet_5s	93.21	94.66	91.85	94.82	93.10	0.8645	50.00
GoogleNet_5s	82.1	88.98	73.33	90.92	80.40	0.6526	50.00
**ResNet50_5s**	**94.92**	**95.45**	**94.33**	**95.50**	**94.89**	**0.8983**	**50.00**
AlexNet_10s	93.50	94.39	92.50	94.50	93.43	0.8701	50.00
GoogleNet_10s	69.17	68.43	71.17	67.17	69.77	0.3836	50.00
ResNet50_10s	93.42	93.49	93.13	93.50	93.41	0.8683	50.00
AlexNet_15s	89.63	90.96	88.00	91.25	89.45	0.7921	50.00
GoogleNet_15s	70.25	73.41	63.50	77.00	68.10	0.4087	50.00
ResNet50_15s	91.25	92.09	90.25	92.25	91.16	0.8251	50.00
AlexNet_20s	89.83	92.83	86.33	93.33	89.46	0.7986	50.00
GoogleNet_20s	67.33	67.69	66.33	68.33	67.00	0.3467	50.00
ResNet50_20s	90.00	92.25	87.33	92.67	89.73	0.8011	50.00

**Table 7:** Comparison of AUC for CNN models.

DL-Models	AUC
AlexNet-5s	0.980
AlexNet-10s	0.981
AlexNet-15s	0.965
AlexNet-20s	0.952
GoogleNet-5s	0.911
GoogleNet-10s	0.764
GoogleNet-15s	0.765
GoogleNet-20s	0.730
**ResNet50-5s**	**0.991**
ResNet50-10s	0.984
ResNet50-15s	0.973
ResNet50-20s	0.966

**Table 8:** Performance comparison of selected studies on PTSD classification.

Author, Year	Dataset Type	Method	Classifier Used	Performance	Strengths	Limitations
Yang et al., 2021 [25]	fMRI	Graph theory + DL	SVM	Accuracy = 71.2%	Combines brain imaging with ML	Requires expensive equipment
Banerjee et al., 2019 [27]	Speech	Frequency feature extraction	DBN + Transfer Learning	Accuracy = 74.99%	Non-invasive and portable	Affected by environment
Schultebraucks et al., 2020 [46]	Video + Audio	Interviews	DNN	AUC = 0.90	Rich multimodal input	Complex preprocessing
Taha et al., 2021 [47]	Pupillometry	STFT	CNN	Accuracy = 80.42%	Objective signal-based method	Specialized hardware needed
Shim et al., 2021 [26]	EEG	Functional connectivity: PLV + graph metrics	SVM	Accuracy = 70.35%, AUC = 0.85	Widely used EEG markers	Sensitive to artifacts
Beykmohammadi et al., 2022 [48]	EEG	CWT	VGG16	Accuracy = 78.93%	Deep features from time–frequency	Moderate accuracy
Bhattacharya et al., 2024 [49]	fMRI	Triplet-based feature learning	Not Stated	Certainty ≥95% for pure class	Novel framework	Unclear metrics
Vali et al., 2025 [50]	Mixed (military, trauma, disaster)	Systematic review/meta-analysis	Random Forest, XGBoost	AUC: 0.745–0.96	Broad scope; high AUCs	-
Portugal et al., 2023 [51]	fMRI	Pattern recognition + regression	GPR	Predicted PTSD symptoms accurately	Biomarkers identified, contextual fMRI	Small sample, limited generalizability
Quatieri et al., 2023 [52]	Speech (PCL-C)	Emotion-based vocal biomarkers	Emotion-Filtered Acoustic Model	AUC = 0.80	Emotion-driven boost in accuracy	Civilian scale, moderate accuracy
Gupta et al., 2023 [53]	Audio-video + questionnaires	ERD estimation	RF, SVM, Logistic Regression	Correlates well with PTSD severity	Explores gender bias, latent traits	Small sample, incomplete metrics
Shahzad et al., 2021 [54]	rs-fMRI	Resting-state fMRI	ANN	Accuracy = 94.5%	High accuracy; regional insights	Expensive, small sample
Josephine et al., 2022 [55]	Speech	Mel spectrogram, emotion recognition	CNN-LSTM	Accuracy = 98.68%	High accuracy, non-invasive	Indirect labels, clinical validation needed
Terpou et al., 2022 [56]	EEG	Spectral decomposition	SVM	Accuracy = 76%	Frequency-specific insight	Moderate accuracy, signal noise
Shim et al., 2022 [57]	EEG	ERP mean amplitude	SVM	Accuracy = 73.33%	Cognitive trait differentiation	One feature, small dataset
**This study**	ECG	CWT (scalogram); statistical features	CNN (AlexNet, GoogLeNet, ResNet); ML (SVM, KNN, Ensemble)	Accuracy = 94.92%, AUC = 0.99	Accessible signal; first to apply ECG in PTSD DL/ML	Multiclass extension needed

## Data Availability

Data are not publicly unavailable due to privacy and ethical restrictions.

## Abbreviations

The following abbreviations are used in this manuscript:

AUC	Area Under the Curve
BW	Baseline Wander
CHD	Coronary Heart Disease
CNN	Convolutional Neural Network
CVD	Cardiovascular Disease
CWT	Continuous Wavelet Transform
DL	Deep Learning
DNN	Deep Neural Network
ECG	Electrocardiogram
ERD	Emotion Regulation Difficulties
ERP	Event-Related Potential
HR	Heart Rate
ERP	Event-Related Potential
HR	Heart Rate
HRV	Heart Rate Variability
KNN	K-Nearest Neighbor
LDA	Linear Discriminant Analysis
MCC	Matthews Correlation Coefficient
ML	Machine Learning
PCL-C	PTSD Check List Civilian
PCI	Phase Locking Values
PLI	Power Line Interference
PTSD	Post-Traumatic Stress Disorder
ResNet	Residual Network
RMS	Root Mean Square
ROC	Receiver Operating Characteristic Curve
SGDM	Stochastic Gradient Descent with Momentum
SINAD	Signal-to-Noise and Distortion Ratio
SNR	Signal-to-Noise Ratio
STD	Standard Deviation
STFT	Short-Time Fourier Transform
SVM	Support Vector Machine
THD	Total Harmonic Distortion
